# Probing and driving of spin and charge states in double quantum dot under the quench

**DOI:** 10.1038/s41598-019-40038-2

**Published:** 2019-02-28

**Authors:** N. S. Maslova, P. I. Arseyev, V. N. Mantsevich

**Affiliations:** 1 Quantum Technology Center and Quantum electronics department, Faculty of Physics, Lomonosov Moscow State University, 119991 Moscow, Russia; 2 P.N. Lebedev Physical Institute RAS, 119991 Moscow, Russia; 3 Russia National Research University Higher School of Economics, 119991 Moscow, Russia; 4 Quantum Technology Center and Semiconductors department, Faculty of Physics, Lomonosov Moscow State University, 119991 Moscow, Russia

## Abstract

We have analyzed theoretically quenched dynamics of correlated double quantum dot (DQD) due to the switching “on” and “off” coupling to reservoirs. The possibility for controllable manipulation of charge and spin states in the double quantum dot was revealed and discussed. The proposed experimental scheme allows to prepare in DQD maximally entangled pure triplet state and to drive it to another entangled singlet state by tuning both applied bias and gate voltage. It was also demonstrated that the symmetry properties of the total system (double quantum dot coupled to electron reservoirs) allow to resolve the initially prepared two-electron states by detecting non-stationary spin-polarized currents flowing in both reservoirs and controlling the residual charge.

## Introduction

Recently the potential of quantum information processing and quantum computation results in numerous proposals of specific material systems for creation and manipulation under spin and charged states in solids^1,2^. One of the key problems in this area is a development of efficient methods of preparation and detection of many electron states with different spin value and orientation^3–9^. Such states are considered to play an important role in modern nanoelectronic devices, for example, spin pumps^10–13^ and turnstiles^14,15^, spin interference devices^16^, quantum dot spin cellular automata^17–19^ and devices for the qubit information^20,21^. Among the most promising candidates for preparation of several electron states with different spin configurations are coupled quantum dots (QDs) - “artificial molecules”. The possibility of QDs integration in a small size quantum circuits deals with careful analysis of relaxation processes and non-stationary effects influence on the electron transport through the dots system^22–31^. Moreover, electronic transport in such systems is strongly influenced by the inter-particle interaction (Coulomb correlations, electron-phonon interaction, the ratio between the QDs coupling and interaction with the reservoir)^32–35^. Electron transport peculiarities through nanoscale systems also depend on the system symmetry properties, geometry of the experimental setup and the way of switching to reservoir^36,37^. As it was shown in^36,37^, spatial symmetry strongly affects the current properties both in stationary and non-stationary cases. Correct interpretation of quantum effects in nanoscale systems provides an opportunity to use them as a basis for high speed electronic and logic devices^38,39^. Consequently, the problem of charge and spin kinetics in correlated low-dimensional systems due to the coupling with reservoir is really vital. For proper treatment of such states, non-stationary currents flowing through QDs systems should be analyzed^40^. Moreover, non-stationary characteristics provide more information about the properties of nanoscale systems comparing to the stationary ones.

Recently a possible physical implementation of parity measurements for electron spins in quantum dots was proposed^41–43^. Proposed schemes should enable free-qubit measurement based quantum computation. Unfortunately detailed analysis of charge and spin states kinetics and controllable switching between different charge and spin configurations in the correlated quantum dots systems has not been performed up to now.

In the present paper we propose a detailed theoretical analysis of the correlated double quantum dot - electronic reservoirs system controllable transfer through intermediate charge and spin configurations from initially prepared (entangled or un-entangled) to final state. Experimental scheme which allows to prepare maximally entangled pure triplet state *T*^0^ and to drive the system to another entangled singlet state *S*^0^ by applied bias and gate voltage tuning is discussed. It is demonstrated that the symmetry properties of the total system (double quantum dot - electronic reservoirs) allow to resolve the initially prepared two-electron states by detection of non-stationary spin-polarized currents in both reservoirs and control under the residual charge.

## Theoretical Model

**Figure 1:**
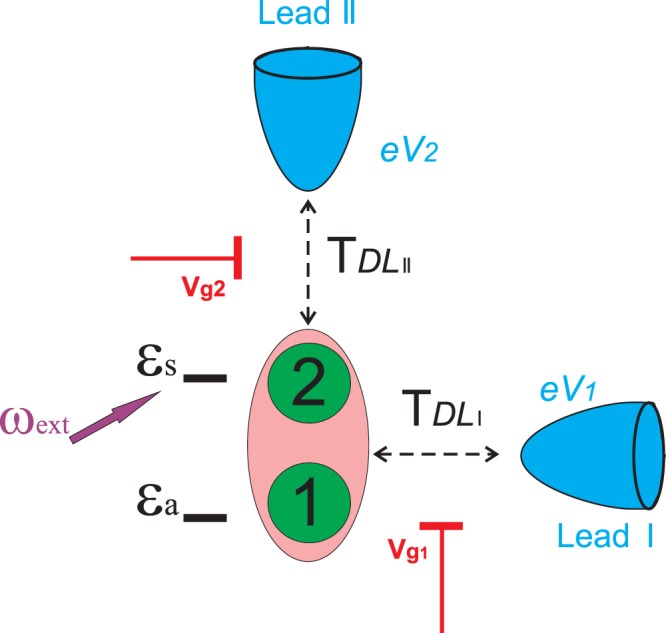
Sketch of the scheme applicable for spin configurations tuning in the correlated double quantum dot (dots are marked by the numbers 1 and 2) localized between the macroscopic leads (reservoirs) I and II. Lead I is symmetrically coupled to the quantum dots system, contrary lead II is coupled directly to only one of the dots. Tunneling transitions between the double quantum dot and the leads could be controlled by means of gate voltages *V*_*g*1(2)_ on gates g1 and g2 and bias voltages *eV*_1(2)_, which are applied directly to the leads. Single electron states with energies *ε*_*S*_(*a*) could be excited by means of external laser pulse with frequency *ω*_*ext*_.

We consider correlated lateral double quantum dot (DQD) coupled to the electron reservoirs (leads) by tunnel barriers (see Fig. 1). The transmission of each tunnel barrier is controlled individually by the voltages on gates *g*1 and *g*2. Strong interdot coupling and weak coupling with the leads occurs. We will not consider vertical double quantum dot structure as in this case characteristics of tunnel barriers are determined by the growth procedure and, consequently, could not be tuned in experiment. The Hamiltonian ${\hat{H}}_{D}$, describing interacting quantum dots reads

1 $${\hat{H}}_{D}=\sum _{l\mathrm{=1,2,}\sigma }{\varepsilon }_{l}{\hat{a}}_{l\sigma }^{+}{\hat{a}}_{l\sigma }+\sum _{l\mathrm{=1,2}}{U}_{l}{\hat{n}}_{ll}^{\sigma }{\hat{n}}_{ll}^{-\sigma }+\sum _{\sigma }T({\hat{a}}_{1\sigma }^{+}{\hat{a}}_{2\sigma }+{\hat{a}}_{2\sigma }^{+}{\hat{a}}_{1\sigma }),$$

where *ε*_*l*_ (*l* = 1, 2) are the spin-degenerate single-electron energy levels and *U*_*l*_ is the on-site Coulomb repulsion for the quantum dots double occupation. Creation/annihilation of an electron with spin *σ* = ±1 within the dot is denoted by operators ${\hat{a}}_{l\sigma }^{+}/{\hat{a}}_{l\sigma }$ and ${\hat{n}}_{ll}^{\sigma }$ is the corresponding occupation number operator. Coupling between the dots is described by tunneling transfer amplitude *T* which is considered to be independent on momentum and spin.

Reservoirs (leads) are modeled by the Hamiltonian:

2 $${\hat{H}}_{res}=\sum _{k\sigma }({\varepsilon }_{k}-e{V}_{1}){\hat{a}}_{k\sigma }^{+}{\hat{a}}_{k\sigma }+\sum _{p\sigma }({\varepsilon }_{p}-e{V}_{2}){\hat{a}}_{p\sigma }^{+}{\hat{a}}_{p\sigma },$$

where operator ${\hat{a}}_{k(p)\sigma }^{+}/{\hat{a}}_{k(p)\sigma }$ creates/annihilates an electron with spin *σ* and momentum *k*(*p*) in the lead. Coupling between the dots and reservoir (lead) I is described by the Hamiltonian:

3 $${\hat{H}}_{tun1}=\sum _{lk\sigma }{T}_{D{L}_{I}}({\hat{a}}_{l\sigma }^{+}{\hat{a}}_{k\sigma }+{\hat{a}}_{k\sigma }^{+}{\hat{a}}_{l\sigma })$$

and interaction with the reservoir (lead) II is given by the Hamiltonian:

4 $${\hat{H}}_{tun2}=\sum _{p\sigma }{T}_{D{L}_{II}}({\hat{a}}_{p\sigma }^{+}{\hat{a}}_{2\sigma }+{\hat{a}}_{2\sigma }^{+}{\hat{a}}_{p\sigma }\mathrm{).}$$

Tunneling transfer amplitudes between continuous spectrum states in the leads and double quantum dot states ${T}_{D{L}_{i}}$ (*i* = *I*, *II*) are independent on momentum and spin. When coupling between QDs exceeds the value of interaction with the reservoirs, one could use the basis of exact eigenfunctions and eigenvalues of coupled QDs without interaction with the leads. In this case all energies of single- and multi-electron states are well known.

Two single electron states basis functions |0↑〉|00〉 and |00〉|0↑〉 correspond to the presence of a single electron with a given spin in each quantum dot. Single electron states wave function reads

5 $${\psi }_{i}^{\sigma }={\mu }_{i}\cdot |0\uparrow \rangle |00\rangle +{\nu }_{i}\cdot |00\rangle |0\uparrow \rangle ,$$

where coefficients *μ*_*i*_ and *ν*_*i*_ are the eigenvectors of the Hamiltonian (1) matrix in the introduced single electron basis. Here index *i* = *S*, *a* and the following ratios between the wave function coefficients occur: for the S-state *μ*_*i*_ = *ν*_*i*_ and for the a-state *μ*_*i*_ = −*ν*_*i*_. For each spin *σ* = +, − one could define single electron states as *S*^±^ and *a*^±^ correspondingly. *S*^±^ are spatially symmetric single electron states with a given spin direction and *a*^±^ correspond to the spatially asymmetric single electron states.

Six two electron states could exist in the DQD system. Among basis functions one could select two functions for the states with the same spin of electrons in each dot *T*^+^ = |↑0〉|↑0〉 and *T*^−^ = |↓0〉|↓0〉. States *T*^+^ and *T*^−^ could be formed only by electrons localized in the different dots. Four basis functions for two electron states with the opposite spins are |↑↓〉|00〉; |00〉|↑↓〉; |↓0〉|0↑〉; |0↑〉|↓0〉. Basis functions |↑↓〉|00〉; |00〉|↑↓〉 correspond to electrons localized in the same quantum dot - the first one or the second one and functions |↓0〉|0↑〉; |0↑〉|↓0〉 describe electrons localized in different dots. Two electron wave function for the state with opposite spins has the form:

6 $${\psi }_{j}^{\sigma -\sigma }={\alpha }_{j}\cdot |\uparrow \downarrow \rangle |00\rangle +{\beta }_{j}\cdot |\downarrow 0\rangle |0\uparrow \rangle +{\gamma }_{j}\cdot |0\uparrow \rangle |\downarrow 0\rangle +{\delta }_{j}\cdot |00\rangle |\uparrow \downarrow \rangle ,$$

where coefficients *α*_*j*_, *β*_*j*_, *γ*_*j*_, *δ*_*j*_ are the eigenvectors of the Hamiltonian (1) matrix in the introduced basis for the opposite spin two electron states. These are low energy singlet *S*^0^ and triplet *T*^0^ states and excited singlet (*S*^0*^) and triplet states (*T*^0*^).

Two three electron states basis functions are |↑↓〉|↑0〉 and |↑0〉|↑↓〉. These functions correspond to the one dot fully occupied by two electrons with opposite spins and only single electron with a given spin in another dot. Corresponding wave function could be written as:

7 $$\begin{array}{rcl}{\psi }_{m}^{\sigma \sigma -\sigma } & = & {p}_{m}\cdot |\uparrow \downarrow \rangle |\uparrow 0\rangle +{q}_{m}\cdot |\uparrow 0\rangle |\uparrow \downarrow \rangle \\ m & = & \pm \mathrm{1,}\end{array}$$

where coefficients *p*_*m*_, *q*_*m*_ are determined by the eigenvectors of the Hamiltonian (1) matrix written in the introduced basis for three electron states.

Finally, single four-electron state exists in the system (both quantum dots are fully occupied) with the wave function

8 $${\psi }_{n}=|\uparrow \downarrow \rangle |\uparrow \downarrow \rangle \mathrm{.}$$

Kinetics of double quantum dot system could be analyzed by means of the pseudo-particles formalism^44,45^, which involves pseudo-particles for each eigenstate of the system. This directly means that the electron operator ${\hat{a}}_{l\sigma }^{+}$ (*l* = 1, 2) is a combination of pseudo-particle operators:

9 $$\begin{array}{rcl}{\hat{a}}_{l\sigma }^{+} & = & \sum _{i}{X}_{i}^{\sigma l}{\hat{f}}_{i\sigma }^{+}\hat{b}+\sum _{ji\sigma }{Y}_{ji}^{\sigma -\sigma l}{\hat{d}}_{j}^{+\sigma -\sigma }{\hat{f}}_{i-\sigma }+\sum _{i\sigma }{Y}_{i}^{\sigma \sigma l}{\hat{d}}^{+\sigma \sigma }{\hat{f}}_{i\sigma }+\sum _{mj\sigma }{Z}_{mj}^{\sigma \sigma -\sigma l}{\hat{\psi }}_{m-\sigma }^{+}{\hat{d}}_{j}^{\sigma -\sigma }\\  &  & +\,\sum _{m\sigma }{Z}_{m}^{\sigma -\sigma -\sigma l}{\hat{\psi }}_{m\sigma }^{+}{\hat{d}}^{-\sigma -\sigma }+\sum _{m\sigma }{{\rm{\Pi }}}_{m}^{\sigma -\sigma -\sigma l}{\hat{\phi }}^{+}{\hat{\psi }}_{m\sigma },\end{array}$$

where ${\hat{f}}_{\sigma }^{+}({\hat{f}}_{\sigma })$ and ${\hat{\psi }}_{\sigma }^{+}({\hat{\psi }}_{\sigma })$ are pseudo-fermion creation (annihilation) operators for electron states with one and three electrons correspondingly. ${\hat{b}}^{+}(\hat{b})$, ${\hat{d}}^{+\sigma }({\hat{d}}^{\sigma })$ and ${\hat{\phi }}^{+}(\hat{\phi })$ are slave boson operators, corresponding to states without electrons, with two electrons or four electrons. Operators ${\hat{\psi }}_{m-\sigma }^{+}$ describe a system configuration with three electrons. Quantities ${X}_{i}^{\sigma l}$, ${Y}_{ji}^{\sigma -\sigma l}$, ${Y}_{i}^{\sigma \sigma l}$, ${Z}_{mj}^{\sigma \sigma -\sigma l}$, ${Z}_{m}^{\sigma -\sigma -\sigma l}$ and ${{\rm{\Pi }}}_{m}^{\sigma -\sigma -\sigma l}$ are matrix elements of the creation operators ${\hat{a}}_{l\sigma }^{+}$ between the states with *n* and *n* + 1 electrons^13^.

Such representation requires the constraint on the possible physical states of the system

10 $${\hat{n}}_{b}+\sum _{i\sigma }{\hat{n}}_{i\sigma }+\sum _{j\sigma \sigma ^{\prime} }{\hat{n}}_{j}^{\sigma \sigma ^{\prime} }+\sum _{m\sigma }{\hat{n}}_{\psi m\sigma }+{\hat{n}}_{\phi }=1,\,.$$

There exists another well known method to analyze the properties of atomic-size devices based on Hubbard operators, which was applied to investigate electron transport through correlated QDs^46,47^. However, pseudo-particle approach seems to be more convenient in some cases as it allows to generalyze Keldysh diagram technique with full account of constraint on the pseudo-particle total occupation^48^. The rules for constructing diagrams for Hubbard operators seems more cumbersome due to the non-trivial commutation relations for these operators.

Coulomb interaction leads to the presence of a gap between the excited double-occupied electron states as well as three- and four-particle states and single- and low energy two-electron states. Consequently, all terms containing operators ${\hat{\phi }}^{+}$ and ${\hat{\psi }}_{m-\sigma }^{+}$ in expressions (8 and 9) could be omitted. Equations for the pseudo-particle occupation numbers ${n}_{i}^{\sigma }$, ${n}_{j}^{\sigma -\sigma }$, ${n}_{j}^{\sigma \sigma }$ and *n*_*b*_ could be derived by averaging equations of motion for the operators and by decoupling the electrons occupation numbers in the double quantum dot system from the reservoir occupation numbers^49,50^. So, considering the constraint on the possible physical states, the following non-stationary system of equations for the pseudo-particle occupation numbers could be written:

11 $$\begin{array}{rcl}\frac{\partial {n}_{j}^{\sigma -\sigma }}{\partial t} & = & -\sum _{i\sigma }\,[{\lambda }_{ji}^{\sigma -\sigma }\mathrm{(1}-{{\rm{\Phi }}}_{k-\sigma }^{ji}){n}_{j}^{\sigma -\sigma }-{\lambda }_{ji}^{\sigma -\sigma }{{\rm{\Phi }}}_{k-\sigma }^{ji}{n}_{i}^{\sigma }],\\ \frac{\partial {n}_{i}^{\sigma }}{\partial t} & = & \sum _{j}\,[{\lambda }_{ji}^{\sigma -\sigma }\mathrm{(1}-{{\rm{\Phi }}}_{k-\sigma }^{ji}){n}_{j}^{\sigma -\sigma }+{\lambda }_{ji}^{\sigma \sigma }\mathrm{(1}-{{\rm{\Phi }}}_{k\sigma }^{ji}){n}_{j}^{\sigma \sigma }]-\sum _{j}\,[{\lambda }_{ji}^{\sigma -\sigma }{{\rm{\Phi }}}_{k-\sigma }^{ji}{n}_{i}^{\sigma }\\  &  & -{\lambda }_{i}\mathrm{(1}-{{\rm{\Phi }}}_{k\sigma }^{i}){n}_{i}^{\sigma }+{\lambda }_{i}{{\rm{\Phi }}}_{k\sigma }^{i}{n}_{b}-{\lambda }_{ji}^{\sigma \sigma }{{\rm{\Phi }}}_{k\sigma }^{ji}{n}_{i}^{\sigma }],\\ \frac{\partial {n}_{b}}{\partial t} & = & \sum _{i\sigma }\,{\lambda }_{i}[{n}_{i}^{\sigma }\mathrm{(1}-{{\rm{\Phi }}}_{k\sigma }^{i})-{{\rm{\Phi }}}_{k\sigma }^{i}{n}_{b}],\\ \frac{\partial {n}_{j}^{\sigma \sigma }}{\partial t} & = & -\sum _{i}\,[{\lambda }_{ji}^{\sigma \sigma }\mathrm{(1}-{{\rm{\Phi }}}_{k\sigma }^{ji}){n}_{j}^{\sigma \sigma }-{\lambda }_{ji}^{\sigma \sigma }{{\rm{\Phi }}}_{k\sigma }^{ji}{n}_{i}^{\sigma }\mathrm{].}\end{array}$$

In the case of symmetric coupling between QDs system and the reservoir (lead) I kinetic coefficients are

12 $$\begin{array}{l}{\lambda }_{i}^{I}={\lambda }_{ji}^{I\sigma \sigma }=2{\gamma }_{I}\cdot |{\mu }_{i}+{\nu }_{i}{|}^{2},\\ {\lambda }_{ji}^{I\sigma -\sigma }=2{\gamma }_{I}\cdot |{\alpha }_{j}{\mu }_{i}+{\beta }_{j}{\nu }_{i}+{\delta }_{j}{\nu }_{i}+{\gamma }_{j}{\mu }_{i}{|}^{2}\mathrm{.}\end{array}$$

In the situation when coupling with the reservoir (lead) II occurs (reservoir II is coupled only with QD 2):

13 $$\begin{array}{rcl}{\lambda }_{i}^{II} & = & 2{\gamma }_{II}\cdot |{\nu }_{i}{|}^{2},\\ {\lambda }_{ji}^{II\sigma -\sigma } & = & 2{\gamma }_{II}\cdot |{\delta }_{j}{\nu }_{i}+{\gamma }_{j}{\mu }_{i}{|}^{2},\\ {\lambda }_{ji}^{II\sigma \sigma } & = & 2{\gamma }_{II}\cdot |{\mu }_{i}{|}^{2},\end{array}$$

where index *i* = *a*, *s* (asymmetric, symmetric single electron state) and the relaxation rate ${\gamma }_{I(II)}=\pi {\nu }_{0}{T}_{DLI(II)}^{2}$ (*ν*_0_ is the electron density of states in the reservoir). Functions ${{\rm{\Phi }}}_{k-\sigma }^{jiX}$ and ${{\rm{\Phi }}}_{k\sigma }^{iX}$ depend on reservoir properties and have the form:

$$\begin{array}{rcl}{{\rm{\Phi }}}_{k-\sigma }^{jiX} & = & \frac{1}{2\pi }i\cdot \int \,d{\varepsilon }_{k}\,{f}_{k}^{X\sigma }({\varepsilon }_{k})\times [\frac{1}{{E}_{j}^{\sigma \sigma ^{\prime} }-{\varepsilon }_{i}+\frac{i{\lambda }_{ji}^{X\sigma -\sigma }}{2}-{\varepsilon }_{k}}-\frac{1}{{E}_{j}^{\sigma \sigma ^{\prime} }-{\varepsilon }_{i}-\frac{i{\lambda }_{ji}^{X\sigma -\sigma }}{2}-{\varepsilon }_{k}}],\\ {{\rm{\Phi }}}_{k\sigma }^{iX} & = & \frac{1}{2\pi }i\cdot \int \,d{\varepsilon }_{k}\,{f}_{k}^{X\sigma }({\varepsilon }_{k})\times [\frac{1}{{\varepsilon }_{i}+\frac{i{\lambda }_{i}^{X}}{2}-{\varepsilon }_{k}}-\frac{1}{{\varepsilon }_{i}-\frac{i{\lambda }_{i}^{X}}{2}-{\varepsilon }_{k}}]\end{array}$$

where *X* = *I*, *II* and ${f}_{k}^{X\sigma }({\varepsilon }_{k})$ is the Fermi distribution function of electrons in the reservoirs (leads). The system of kinetic equations (11) should be solved with the initial conditions for each reservoir pseudo-particle occupation number. For symmetric coupling to the reservoir I (${\lambda }_{a}^{I}=0$), system of equations (11) could be solved as two independent systems of equations. One of them contains equations for the occupation numbers ${n}_{{T}^{0}}$ and ${n}_{{a}^{\pm }}$ and another one describes dynamics of the occupation numbers ${n}_{{T}^{\pm }}$, ${n}_{{S}^{0}}$, ${n}_{{S}^{\pm }}$ and *n*_*b*_. It is also reasonable to group the initial conditions for each system and determine them as *M*(0) and *L*(0). Due to the constraint on the possible physical states one has *M*(0) + *L*(0) = 1. Consequently, the corresponding initial conditions are

14 $$\begin{array}{rcl}M\mathrm{(0)} & = & {n}_{{T}^{0}}\mathrm{(0)}+2{n}_{{a}^{\pm }}\mathrm{(0),}\\ L\mathrm{(0)} & = & 2{n}_{{T}^{\pm }}\mathrm{(0)}+{n}_{{S}^{0}}\mathrm{(0)}+2{n}_{{S}^{\pm }}\mathrm{(0)}+{n}_{b}\mathrm{(0).}\end{array}$$

In the case of identical QDs and initial two-electron state (which can be also a mixed state), the pseudo-particle occupation numbers time evolution could be obtained from Eq. (11):

15 $${n}_{i}(t)={n}_{i}^{st}+[{n}_{i}\mathrm{(0)}-{n}_{i}^{st}]\cdot {e}^{-{\lambda }_{\xi }^{I}t},$$

where *i* = *T*^0^, *S*^0^, *a*^±^, *S*^±^, *T*^±^, *b* and *ξ* = *T*^0^ for *i* = *T*^0^, *a*^±^. Parameter *ξ* = *S*^0^ for *i* = *S*^0^, *S*^±^, *T*^±^, *b*.

Relaxation rate

16 $${\lambda }_{{T}^{0}}^{I}=4{\gamma }_{I}\cdot (1-\frac{{{\rm{\Phi }}}_{k-\sigma }^{{T}^{0}a}I}{2})$$

determines time evolution of occupation numbers in the two electron triplet state *T*^0^ and asymmetric single electron state *a*. Relaxation rate

17 $${\lambda }_{{S}^{0}}^{I}=8{\gamma }_{I}\cdot |\alpha +\beta {|}^{2}\cdot (1-\frac{{{\rm{\Phi }}}_{k-\sigma }^{{S}^{0}S}I}{2})$$

describes time evolution of occupation numbers in the two electron singlet state *S*^0^. Stationary values of partial pseudo-particles occupation numbers are:

18 $$\begin{array}{rcl}{n}_{{T}^{\pm }}^{st} & = & \frac{L\mathrm{(0)}}{Z}\cdot {{\rm{\Phi }}}_{k\sigma }^{S}{{\rm{\Phi }}}_{k-\sigma }^{{T}^{\pm }{S}^{\pm }}\mathrm{(1}-{{\rm{\Phi }}}_{k-\sigma }^{{S}^{0}S}),\\ {n}_{{S}^{0}}^{st} & = & \frac{L\mathrm{(0)}}{Z}\cdot {{\rm{\Phi }}}_{k\sigma }^{S}{{\rm{\Phi }}}_{k-\sigma }^{{S}^{0}S}\mathrm{(1}-{{\rm{\Phi }}}_{k-\sigma }^{{T}^{\pm }{S}^{\pm }}),\\ {n}_{{S}^{\pm }}^{st} & = & \frac{L\mathrm{(0)}}{Z}\cdot {{\rm{\Phi }}}_{k\sigma }^{S}(1-{{\rm{\Phi }}}_{k-\sigma }^{{S}^{0}S})(1-{{\rm{\Phi }}}_{k-\sigma }^{{T}^{\pm }{S}^{\pm }}),\\ {n}_{b}^{st} & = & \frac{L\mathrm{(0)}}{Z}\cdot (1-{{\rm{\Phi }}}_{k\sigma }^{S})(1-{{\rm{\Phi }}}_{k-\sigma }^{{S}^{0}S})(1-{{\rm{\Phi }}}_{k-\sigma }^{{T}^{\pm }{S}^{\pm }}),\\ {n}_{{T}^{0}}^{st} & = & M\mathrm{(0)}\cdot \frac{{{\rm{\Phi }}}_{k-\sigma }^{{T}^{0}a}}{2-{{\rm{\Phi }}}_{k-\sigma }^{{T}^{0}a}},\\ {n}_{{a}^{\pm }}^{st} & = & M\mathrm{(0)}\cdot \frac{1-{{\rm{\Phi }}}_{k-\sigma }^{{T}^{0}a}}{2-{{\rm{\Phi }}}_{k-\sigma }^{{T}^{0}a}},\end{array}$$

and

19 $$\begin{array}{rcl}Z & = & \mathrm{2(1}-{{\rm{\Phi }}}_{k-\sigma }^{{S}^{0}S}\mathrm{)(1}-{{\rm{\Phi }}}_{k-\sigma }^{{T}^{\pm }{S}^{\pm }}){{\rm{\Phi }}}_{k\sigma }^{S}+{{\rm{\Phi }}}_{k-\sigma }^{{S}^{0}S}{{\rm{\Phi }}}_{k\sigma }^{S}\mathrm{(1}-{{\rm{\Phi }}}_{k-\sigma }^{{T}^{\pm }{S}^{\pm }})\\  &  & +\,2{{\rm{\Phi }}}_{k-\sigma }^{{T}^{\pm }{S}^{\pm }}{{\rm{\Phi }}}_{k\sigma }^{S}\mathrm{(1}-{{\rm{\Phi }}}_{k\sigma }^{{S}^{0}S})+\mathrm{(1}-{{\rm{\Phi }}}_{k\sigma }^{S}\mathrm{)(1}-{{\rm{\Phi }}}_{k-\sigma }^{{T}^{\pm }{S}^{\pm }}\mathrm{)(1}-{{\rm{\Phi }}}_{k-\sigma }^{{S}^{0}S}),\end{array}$$

where functions ${{\rm{\Phi }}}_{k-\sigma }^{ji}$ and ${{\rm{\Phi }}}_{k\sigma }^{i}$ in Eqs (18–19) correspond to the reservoir (lead) I.

The regime of weak coupling between double quantum dot system and reservoirs $(\frac{|{\varepsilon }_{S(a)}-{\varepsilon }_{F}|}{\gamma }\gg 1)$ and particular position of the Fermi level in the lead I between the single-electron states with energies (*ε*_*S*_ < *E*_*F*_ and *ε*_*a*_ > *E*_*F*_) means that the values of the reservoir functions ${{\rm{\Phi }}}_{k\sigma }^{S}$ and ${{\rm{\Phi }}}_{k-\sigma }^{{T}^{0}a}$ are very close to unity, while the values of the functions ${{\rm{\Phi }}}_{k-\sigma }^{{T}^{\pm }{S}^{\pm }}$, ${{\rm{\Phi }}}_{k\sigma }^{a}$ asymptotically approach to zero (see Eq. 14). The stationary state pseudo-particle occupation numbers are determined by the reservoir occupation functions ${{\rm{\Phi }}}_{k-\sigma }^{{S}^{0}S}$ and ${{\rm{\Phi }}}_{k}^{{T}^{0}a}$, which depend on the energies ${E}_{{S}^{0}}-{\varepsilon }_{S}$ and ${E}_{{T}^{0}}-{\varepsilon }_{a}$ correspondingly. So, the triplet state *T*^0^ doesn’t decay for any value of Coulomb interaction, as the decay process is governed by the function ${{\rm{\Phi }}}_{k-\sigma }^{{T}^{0}a}$, which is equal to unity. For condition ${E}_{{S}^{0}}-{\varepsilon }_{S} < {E}_{F} < {E}_{{T}^{0}({T}^{\pm })}-{\varepsilon }_{S}$ and small relaxation rate *γ*_*I*_ occupation numbers ${n}_{{S}^{0}}$ could be also close to unity. The situation differs when the Fermi level lies far below the single electron states *ε*_*S*(*a*)_. Then, stationary occupation of the triplet state *T*^0^ turns to zero [see Eqs (18–19)] and the stationary occupation of single electron states ${n}_{{a}^{\pm }}^{st}$ turns to $\frac{1}{2}$. So, the residual charge *e* remains in the quantum dots system.

This situation changes when only one of the quantum dots (2) is coupled to the reservoir II. In this case sequential coupling of QDs system to reservoir occurs. Consequently, there are no symmetry governed selection rules for electron transitions from localized states in the QDs system to the reservoir II. So, for the Fermi level situated far below all the transfer energies between the QDs states with *n* and *n* ± 1 electrons (reservoir is “empty”) all the stationary values of the pseudo-particle occupation numbers except *n*_*b*_ turns to zero, *n*_*b*_ turns to unity.

## Main Results and Discussion

### Controllable manipulation of electron states in the correlated double quantum dots

We would like to analyzed the situation when reservoir I is symmetrically coupled to the double quantum dot and the reservoir II is interacting only with the dot 2. DQD is separated from the leads by potential barriers whose heights could be varied via gate electrodes. There is no need to control the barriers heights with high precision, the only need is to switch “on” or “off” tunneling between the DQD and the reservoirs. Results, obtained in the previous section open a possibility to propose the scheme for the controllable spin and charge manipulation in the correlated DQD by means of applied bias voltage and gate voltage changing. On the first stage interaction between the dots and the reservoirs is switched “off”. Strong coupling between the dots results in the quantum mechanical coupling between the electron states in the dots and in this case single electron states energies are

20 $${\varepsilon }_{a(s)}=\frac{{\varepsilon }_{1}+{\varepsilon }_{2}}{2}\pm \sqrt{\frac{{({\varepsilon }_{1}-{\varepsilon }_{2})}^{2}}{4}+{T}^{2}}\mathrm{.}$$

**Figure 2:**
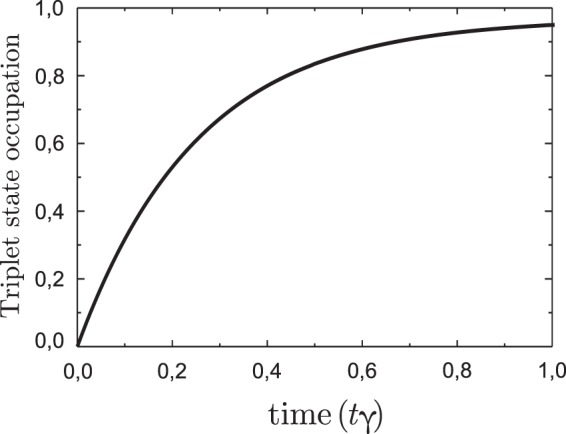
Time evolution of the triplet state filling ${n}_{{T}^{0}}$. The parameters are *U*/*T* = 6.5, *ε*_1_/*γ* = *ε*_2_/*γ* = −5.0, *T*/*γ* = 15 and *γ* = 1. The initial conditions are *n*_*a*_ = 0.5, ${n}_{{S}^{0}}\mathrm{(0)}={n}_{{T}^{0}}\mathrm{(0)}={n}_{{T}^{\pm }}\mathrm{(0)}=0.0$.

**Figure 3:**
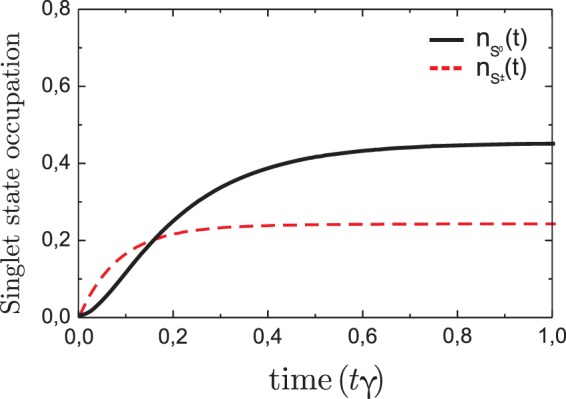
Time evolution of partial pseudo particle occupation numbers ${n}_{{S}^{0}}(t)$ (black solid line) and ${n}_{{S}^{\pm }}(t)$ (red dashed line), which reveals filling of the singlet state. The parameters are *U*/*T* = 6.5, *ε*_1_/*γ* = *ε*_2_/*γ* = −5.0, *T*/*γ* = 15 and *γ* = 1. The initial conditions are *n*_*b*_ = 1.0, ${n}_{{S}^{0}}\mathrm{(0)}={n}_{{T}^{0}}\mathrm{(0)}={n}_{{T}^{\pm }}\mathrm{(0)}=0.0$.

Single electron state with the energy *ε*_*a*_ should be initially excited, for example, by the external laser pulse. The symmetric coupling to the reservoir I is switched “on” at particular time moment by lowering the potential barrier by means of the gate voltage *V*_*g*1_. Simultaneously the gate voltage *V*_*g*2_ has the value which uncouples quantum dots from the reservoir II. Tuning bias voltage applied to the reservoir I one could drive the position of the reservoir Fermi level. If the reservoir I Fermi level *E*_*F*_ is localized between the single electron energy states *ε*_*S*_ and *ε*_*a*_ filling of the triplet state *T*^0^ becomes close to unity (see Fig. 2). Further tunneling between the reservoir I and DQD is switched “off” by means of the gate voltage *V*_*g*1_ and interaction with the “empty” reservoir II is switched “on” by means of the tunnel barrier lowering by changing the gate voltage *V*_*g*2_ (filling of the reservoirs can be tuned by applied bias values *eV*_*i*_). It means that the occupation of the triplet state *T*^0^ turns to zero and all other electron states become empty. So, in the pseudo particle representation the only occupied stationary state is *n*_*b*_ = 1. The last step of the scheme deals with switching “off” interaction with the reservoir II by increasing the barrier by means of the gate voltage *V*_*g*2_ and switching “on” interaction with the reservoir I by means of the gate voltage *V*_*g*1_. This could result in formation of the occupied singlet *S*^0^ state for particular value of the applied bias *eV*_1_ (see Fig. 3). Filling of the *S*^±^ states occurs due to the energies ${\varepsilon }_{{S}^{\pm }}$ localization below the Fermi level of the lead I (${\varepsilon }_{{S}^{\pm }} < {E}_{{F}_{1}}$). The fulfillment of this condition is controlled by the bias voltage *eV*_1_. Singlet *S*^0^ state fills due to the fulfillment of the following relation between the energies in the system: ${E}_{{S}^{0}}-{\varepsilon }_{S} < {E}_{{F}_{1}}$. This ratio is valid if ${\varepsilon }_{0}+T-\frac{{T}^{2}}{{U}_{l}} < {E}_{{F}_{1}}$. So, occupation of singlet states *S*^0^ and *S*^±^ increases simultaneously, but occupation of *S*^0^ state usually exceeds occupation of *S*^±^ states. The triplet states *T*^±^ occupation doesn’t increase because the difference between the energies of the triplet states *T*^±^ and singlet states *S*^±^ exceeds the value of Fermi energy (${E}_{{T}^{\pm }}-{E}_{{S}^{\pm }} > {E}_{{F}_{1}}$) and there are no electrons with proper energies in the reservoir.

**Figure 4:**
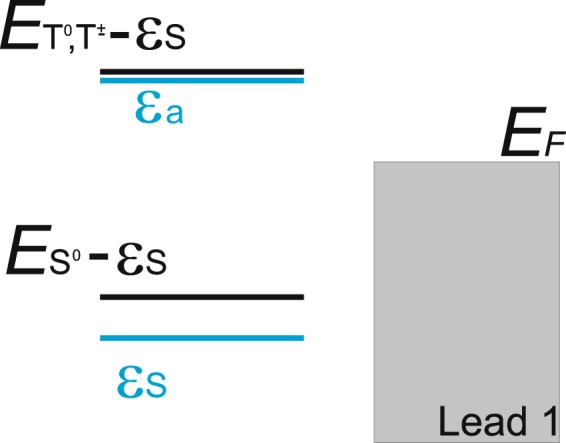
Scheme of the energy levels illustrating filling of the singlet *S*^0^ state.

We would like to mention that the triplet states *T*^0^, *T*^±^ do not fill during the system time evolution and the singlet state *S*^0^ reveals growth of the occupation because for the chosen value of bias voltage in the leads (see Fig. 4) the following relations between the energies in the system occur: ${E}_{{T}^{0},{T}^{\pm }}-{\varepsilon }_{S} > {E}_{{F}_{1}}$ and ${E}_{{S}^{0}}-{\varepsilon }_{S} < {E}_{{F}_{1}}$. This relations mean the absence of conduction electrons in the lead I with the energies close to ${E}_{{T}^{0},{T}^{\pm }}-{\varepsilon }_{S}$; contrary there exist electrons with energies in the vicinity of ${E}_{{S}^{0}}-{\varepsilon }_{S}$ in the lead I. Conduction electrons states with the energies ${E}_{T}^{\pm }-{\varepsilon }_{S}$ are nearly empty as Fermi level is localized between the single electron energy levels *ε*_*S*(*a*)_ and, consequently, *T*^±^ states could not be occupied. Triplet state occupation *T*^0^ doesn’t increase due to the selection rules which restrict transitions between singlet *S*^±^ states and triplet *T*^0^ state. Single electron states *a*^±^ filling for the symmetric coupling of DQD with the reservoir is also forbidden by the selection rules. Meanwhile, filling of the triplet state *T*^0^ is possible only from the single electron *a*^±^ state.

The proposed scheme allows to prepare controllable the maximally entangled pure triplet *T*^0^ state and to drive the system from the *T*^0^ state to the another entangled singlet *S*^0^ state by applied bias and gate voltage changing. One could also follow time evolution of the degree of entanglement by analyzing the behavior of two-electron correlation functions^51^.

### Diagnostics of two electron states in the correlated quantum dots

**Figure 5:**
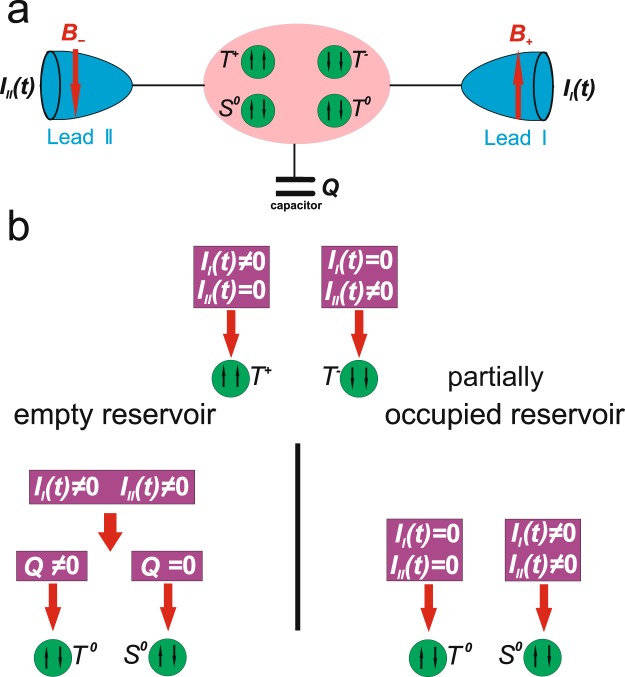
Sketch of the measurement setup (**a**)and logic diagram (**b**), which enable to resolve initial many particle electron states with different spin orientation for both “empty” and “partially occupied” reservoirs.

DQD system is a promising candidate for the diagnostics of initially prepared two-electron states. As it was shown in Section 1.1 sequential combination of symmetric and asymmetric coupling to the leads results in the controllable manipulation of spin and charge states. The question of interest is the possibility to probe prepared states. For this goal symmetrical coupling scheme to two reservoirs is more consistent as it allows to distinguish initially prepared two-electron states (*T*^0^, *T*^±^ or *S*^0^) by analyzing non-stationary spin-polarized currents in the both reservoirs and controlling residual charge in the QDQ. It is convenient to apply symmetric coupling for diagnostics as in this case single electron states *a*^±^ are uncoupled from the reservoir and residual charge could be localized in them. This doesn’t happen for symmetric coupling. Diagnostics scheme differs depending on the position of the Fermi levels in the reservoirs with respect to the DQD single electron energy levels. Let us distinguish two possible reservoir states: “empty” reservoir and “partially occupied” reservoir. “Empty” means that reservoir Fermi level lies well below the DQD single electron states. “Partially occupied” reservoir correspond to the situation when Fermi level lies between the DQD single electron states *ε*_*a*_ < *E*_*F*_ < *ε*_*s*_ and $\frac{|{E}_{F}-\varepsilon a(S)|}{{\gamma }_{I(II)}}\gg 1$. To identify the triplet *T*^±^ states one should also apply oppositely directed magnetic fields to the reservoirs (see Fig. 5).

One should start from the diagnostics of the triplet states *T*^+^ and *T*^−^ as they could be resolved only by the measurements of the non-stationary spin-polarized currents in the reservoirs. Procedure for the *T*^+^ and *T*^−^ resolution is the same for “empty” and “partially occupied” reservoirs. The absence of non-stationary current in the left lead (*I*_*II*_ = 0) and the presence of non-stationary current in the right lead *I*_*I*_ means that triplet state *T*^+^ was an initial one. The opposite situation corresponds to the *T*^−^ initial state (see logic diagram in the Fig. 5). Further diagnostics of the singlet *S*^0^ and triplet *T*^0^ states could be performed. In addition to the the non-stationary currents measurements one should analyze the residual charge. Moreover, measurements procedure differs for the “empty” and “partially occupied” reservoirs. For “empty” reservoirs singlet *S*^0^ and triplet *T*^0^ states could be resolved by means of control under residual charge *Q* just after the registration of the non-stationary current pulses (*I*_*I*_ ≠ 0 and *I*_*II*_ ≠ 0) in both leads. The absence of the residual charge *Q* = 0 corresponds to the singlet *S*^0^ initial state. The presence of residual charge *Q* ≠ 0 means triplet *T*^0^ initial state (see logic diagram in the Fig. 5). For “partially occupied” reservoirs *T*^0^ and *S*^0^ states could be identified by simultaneous registration of non-stationary spin- polarized currents in the both leads. The absence of currents in the both leads means that the initial state was a triplet *T*^0^ state which does not decay. The presence of non-stationary spin-polarized currents in the both leads corresponds to the initial singlet *S*^0^ state (see logic diagram in the Fig. 5).

## Conclusion

In the present paper we demonstrated the possibility for controllable manipulation of the spin and charge states in the correlated quantum dots coupled to the reservoirs. This possibility is based on the fundamental (symmetry) properties of localized two electron states quenched dynamics due to the switching “on” and “off” coupling to the reservoirs. We proposed experimental scheme which allows to prepare maximally entangled pure triplet state *T*^0^ and to drive the system to the another entangled singlet state *S*^0^ by applied bias and gate voltage changing. One could also follow the degree of entanglement changing during the system time evolution by analyzing the behavior of two-electron correlation functions. Moreover, the symmetry properties of the DQD - electron reservoirs system allow to distinguish the initially prepared two-electron state by analyzing non stationary spin polarized currents in the both reservoirs and control under residual charge.
